# Four plant defensins from an indigenous South African Brassicaceae species display divergent activities against two test pathogens despite high sequence similarity in the encoding genes

**DOI:** 10.1186/1756-0500-4-459

**Published:** 2011-10-28

**Authors:** Abré de Beer, Melané A Vivier

**Affiliations:** 1 Institute for Wine Biotechnology, Department of Oenology and Viticulture, Faculty of AgriSciences, Stellenbosch University, Stellenbosch 7600, South Africa

## Abstract

**Background:**

Plant defensins are an important component of the innate defence system of plants where they form protective antimicrobial barriers between tissue types of plant organs as well as around seeds. These peptides also have other activities that are important for agricultural applications as well as the medical sector. Amongst the numerous plant peptides isolated from a variety of plant species, a significant number of promising defensins have been isolated from Brassicaceae species. Here we report on the isolation and characterization of four defensins from *Heliophila coronopifolia*, a native South African Brassicaceae species.

**Results:**

Four defensin genes (*Hc-AFP1*-*4) *were isolated with a homology based PCR strategy. Analysis of the deduced amino acid sequences showed that the peptides were 72% similar and grouped closest to defensins isolated from other Brassicaceae species. The Hc-AFP1 and 3 peptides shared high homology (94%) and formed a unique grouping in the Brassicaceae defensins, whereas Hc-AFP2 and 4 formed a second homology grouping with defensins from *Arabidopsis *and *Raphanus*. Homology modelling showed that the few amino acids that differed between the four peptides had an effect on the surface properties of the defensins, specifically in the alpha-helix and the loop connecting the second and third beta-strands. These areas are implicated in determining differential activities of defensins. Comparing the activities after recombinant production of the peptides, Hc-AFP2 and 4 had IC_50 _values of 5-20 μg ml^-1 ^against two test pathogens, whereas Hc-AFP1 and 3 were less active. The activity against *Botrytis cinerea *was associated with membrane permeabilization, hyper-branching, biomass reduction and even lytic activity. In contrast, only Hc-AFP2 and 4 caused membrane permeabilization and severe hyper-branching against the wilting pathogen *Fusarium solani*, while Hc-AFP1 and 3 had a mild morphogenetic effect on the fungus, without any indication of membrane activity. The peptides have a tissue-specific expression pattern since differential gene expression was observed in the native host. *Hc-AFP1 *and *3 *expressed in mature leaves, stems and flowers, whereas *Hc-AFP2 *and *4 *exclusively expressed in seedpods and seeds.

**Conclusions:**

Two novel Brassicaceae defensin sequences were isolated amongst a group of four defensin encoding genes from the indigenous South African plant *H. coronopifolia*. All four peptides were active against two test pathogens, but displayed differential activities and modes of action. The expression patterns of the peptide encoding genes suggest a role in protecting either vegetative or reproductive structures in the native host against pathogen attack, or roles in unknown developmental and physiological processes in these tissues, as was shown with other defensins.

## Background

Plants have developed complex defence systems to protect them against a multitude of plant pathogens [1-8]. These defence systems consists of an array of both chemical and biochemical substances that protect the plant against colonization and subsequent spread of disease and can broadly be divided into the innate and active defence responses [7,9-13]. The innate defence responses play an important role in establishing preformed barriers of defence to prevent colonization by pathogens. Antimicrobial peptides (AMPs) are an important component of the innate defence response. They are small, mostly basic peptides that range in size from 2-9 kDa and have been classified into nine groups. Plant defensins [10,14-21], thionins [22-27] and lipid transfer proteins [28-34] are the best characterized of these nine groups.

Plant defensins are small, basic, heat stable peptides with a conserved tertiary structure that consists of a single α-helix and three anti-parallel β-strands [17,35-37]. The defensin tertiary structure is internally stabilized by disulphide bridges linking the α-helix to two of the β-strands to form a structure know as the cysteine stabilizing motif, a conserved motif identified in AMPs isolated from various prokaryotes and higher eukaryotes [38-41]. In addition to the cysteine stabilizing motif two additional conserved motives have been identified in the plant defensin structure, namely the α-core, encompassing the loop connecting the first β-strand and α-helix and the γ-core containing the all important hairpin loop connecting β-strand 2 and 3 (Lβ_2_β_3_). Notwithstanding this conserved tertiary structure, plant defensins share very little homology at amino acid level. It is however this variability in primary amino acid sequence that contributes to the different biological functions that have been attributed to these peptides, where a single amino acid can change the spectrum of activity exhibited by closely related defensin peptides.

The role of plant defensins in the preformed defence of plants is well documented. They play an important role in the protection of germinating plant seeds, developing seedlings and reproductive structures of plants [42-44] and have been isolated from roots [44-46], vegetative tissues and reproductive structures such as flowers and fruits [45,47-55]. The majority of characterized plant defensins show a constitutive pattern of expression, with an induction in expression in response to pathogen attack, wounding and some abiotic stresses [20,44-46]. Recently it was shown that pathogen-induced expression of *Arabidopsis *plant defensins is dependent on ENHANCED DISEASE RESISTANCE1 (EDR1), which interferes with the repressor function of *MYC2 *allowing for defensin gene expression [56]. Some defensins, however, show a strict tissue-specific and developmentally regulated pattern of expression [47,50,54,57,58] which in some cases were linked to specific biological functions other than plant defence, as was demonstrated for the defensins from tomato and maize that play a role during pollination [50,57].

Plant defensins are best known for their antimicrobial activity against a broad spectrum of plant pathogens that include bacteria [59,60], yeast [61-64], oomycetes [65,66] and necrotrophic pathogens [47,61,64,65,67-71]. In addition to these strong antimicrobial activities that established them as important agricultural biotechnology targets, some members also show activities important for medical applications, including protease inhibitory activity [23,72], anti cancer activity [61,73] and HIV inhibition [61,74-76]. Other agriculturally important activities include insecticidal activity [35,36,77,78], activity against parasitic plants [79] and heavy metal tolerance [80].

The isolation and characterization of a wide range of defensin peptides are crucial for the continued development of economically and medically important products. Analysis of the sequenced plant genomes revealed that defensins are present as multigene families and are overrepresented in the genomes of some plants species [46,81]. With the wealth of defensin nucleotide sequences available, strategies of gene isolation coupled with recombinant production are increasingly been used for the characterization of closely related plant defensin peptides.

This work describes the successful isolation of four plant defensin genes from the South African Brassicaceae species *Heliophila coronopifolia*. An isolation strategy based on the sequence homology that exists within the nucleotides encoding the signal peptides of defensins from domesticated Brassicaceae species was used to isolate four defensin sequences, of which two were shown to be novel for Brassicaceae defensins. Each of the defensin peptide was successfully purified through recombinant production in *Escherichia coli *and characterized for their activity and mode of action against two test pathogens. These results as well as expression analysis in the host showed that the four peptides have differential expression patterns in vegetative and reproductive organs, as well as differential activities and modes of inhibition under the conditions tested. In addition, the divergence in structural motifs and surface properties observed for these peptides provide interest to study structure-activity determinants in these peptides.

## Results

### Isolation and *in silico *characterization of the Hc-AFP encoding sequences

**Figure 1:**
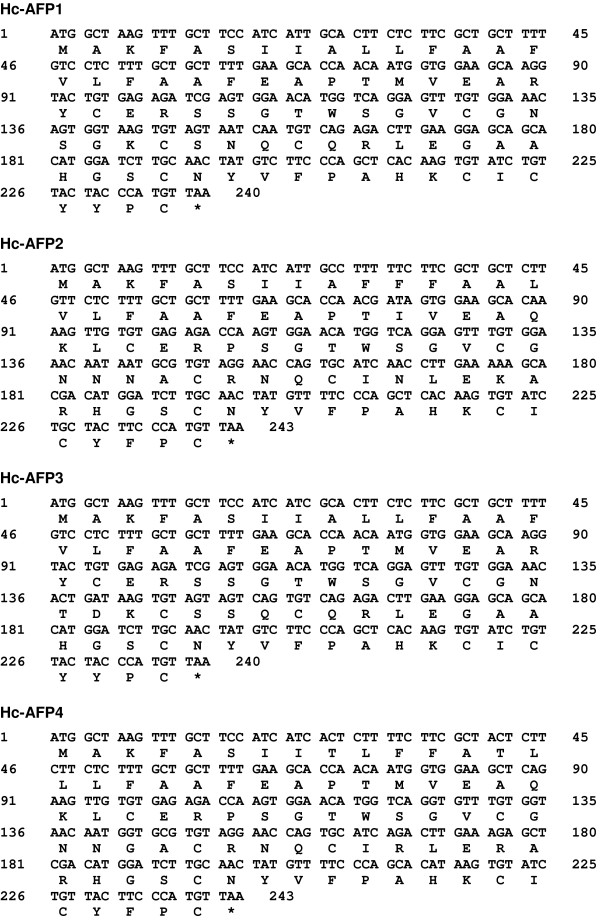
**The cDNA sequences encoding for *Hc-AFPs*, isolated by PCR from cDNA generated from *H. coronopifolia *stem, leaf, flower, siliques and seed tissue**. The Hc-AFP defensins are encoded within the first 240 nucleotides for *Hc-AFP1*and *3 *and the first 243 nucleotides for *Hc-AFP2 *and *4*. The deduced amino acid sequence encoded by each gene is indicated beneath its represented sequence.

PCR-based isolation of cDNA from *H. coronopifolia *tissues allowed for the isolation of four putative defensin sequences ranging between 426 bp and 468 bp, containing open reading frames of 240 and 243 bp, respectively. TBLASTN analysis of the nucleotide sequences showed homology to sequences encoding for the super family of plant antifungal peptides known as plant defensins. The isolated gene sequences were thus termed *H. coronopifolia antifungal peptide 1 *to *4 *(*Hc-AFP 1 *- *4*) (Figure 1).

Analysis of the deduced amino acid sequences showed that *Hc-AFP1 *and *3 *encode for 80 amino acid peptides, whereas *Hc-AFP2 *and *4 *encode for 81 amino acid peptides (Figure 1). SignalP results showed that the first 30 amino acids of each peptide encode for a signal peptide followed by a 50 amino acid mature peptide for Hc-AFP1 and 3 and a 51 amino acid mature peptide for Hc-AFP2 and 4 (Table 1). The peptide parameters obtained from the Expasy-Compute pI/Mw tool (Table 1) showed that the peptides had predicted mono-isotopic masses ranging between 5.48 and 5.73 kDa and are highly basic with isoelectric points above 8.2.

**Table 1:** Peptide parameters of the newly isolated Hc-AFP defensin peptides

Defensin	Signal peptide (amino acids)	Mature peptide (amino acids)	MW (Da)	pI	Charge at pH7
Hc-AFP1	1-29	30-80	5479.32	8.50	3.2

Hc-AFP2	1-29	30-81	5718.31	8.73	4.2

Hc-AFP3	1-29	30-80	5524.33	8.20	2.2

Hc-AFP4	1-29	30-81	5731.61	8.94	5.2

**Figure 2:**
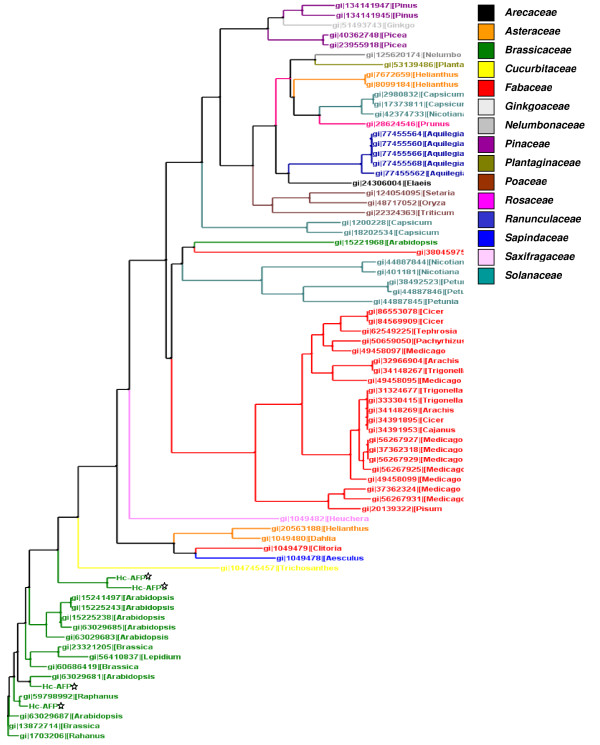
**The phylogenetic relationship of the newly isolated Hc-AFPs with members of the plant defensin super family**. The deduced amino acid sequences of the newly isolated defensins (indicated with *) were aligned in ClustalX with other members of the defensin super family isolated from various plant genera. The tree was created in Arbodraw. The newly isolated defensins showed the closest relation to defensin peptides isolated from other Brassicaceae species.

**Figure 3:**
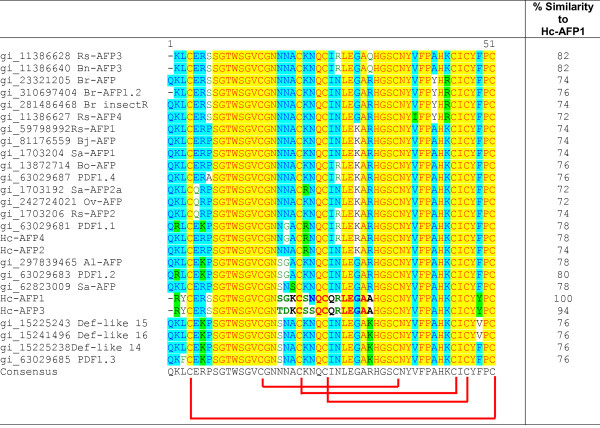
**Alignment of the mature region of the Hc-AFP peptides with members of the plant defensin family isolated from Brassicaceae species**. The percentage similarity compared to Hc-AFP1 is indicated in the last column. *Arabidopsis halleri*: [GenBank 63029681, 63029683, 63029685, 63029687]; *Arabidopsis lyrata*: [GenBank 297839465]; *Arabidopsis thaliana*: [GenBank 18410943, 15225243, 15241496, 15225238]; *Brassica napus*: [GenBank 11386640]; *Brassica juncea*: [GenBank 81176559]; *Brassica oleracea*: [Genbank 13872714]; *Brassica rapa*: [GenBank 23321205, 310697404, 281486468]; *Orychophragmus violaceus*: [GenBank 242724021]; *Raphanus sativa*: [GenBank 59798992, 1703206, 1138662811386627]; *Sinapsis alba*: [GenBank 1703192, 170320462823009]. The disulphide bridge pattern, common to all plant defensins, is indicated in red.

Alignment analysis of the deduced amino acid sequences revealed that the newly isolated *H. coronopifolia *defensins shared the highest homology with defensins isolated from other members of the Brassicaceae family (Figure 2). Disulphide-bridge analyses conducted on the Hc-AFP peptides revealed that they share a disulphide bridge pattern common to all plant defensins (Figure 3). Further comparison of Hc-AFPs with members of the Brassicaceae defensins (Figures 2 and 3) revealed that Hc-AFP1 shared the closest homology to Hc-AFP3 at 94% similarity and *Rs-AFP3 *from *Raphanus sativa *at 82% similarity, whereas Hc-AFP2 showed the greatest homology to the defensins isolated from *Sinapsis alba *and *R. sativa *(Rs-AFP2) at 98% similarity (Figures 2 and 3). Hc-AFP4 was more closely related to PDF1.1 from *A. halleri*, a defensin proposed to play a role in the zinc tolerance of *A. halleri*.

**Figure 4:**
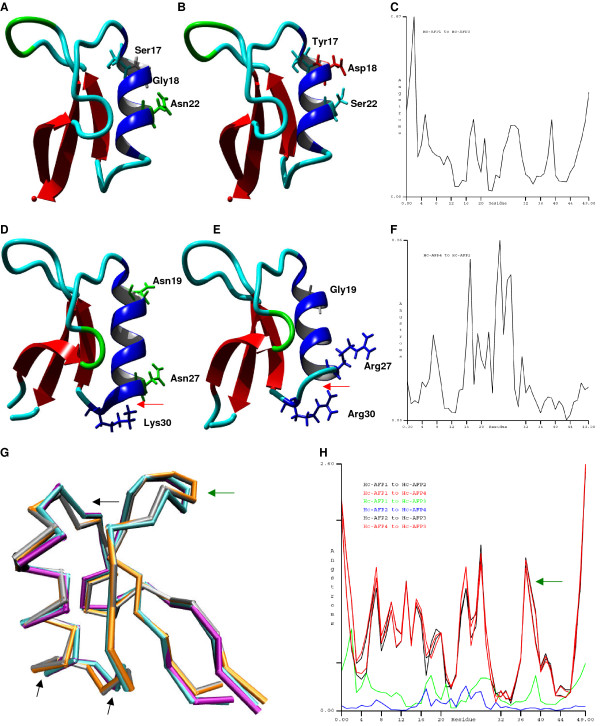
**Homology models of *H. coronopifolia *defensins depicting the amino acid differences observed in the alignment of the deduced amino acid sequences**. (**A**) Hc-AFP1, (**B**) Hc-AFP3, (**D**) Hc-AFP2, (**E **) Hc-AFP4, (**C **and **F**) RMSD analysis of Hc-AFP1 and 3 and Hc-AFP2 and 4 respectively. (**G**) Structural alignment of the backbones of Hc-AFP1-4 (**H**) RMSD analysis of the structural alignment of Hc-AFP1-4. Models were created using Rs-AFP1 (Protein Data Bank: 1AYJ) as template. Acidic residues are indicated in red, basic in blue and polar residues in green. The black arrows indicate significant differences in structure between Hc-AFP1 and 3 (Group 1) and Hc-AFP2 and 4 (Group 2). The green arrow indicates the difference in presentation of the loop connecting β-strand 2 and 3.

Analysis of homology models obtained for the different Hc-AFPs in combination with the alignment analysis of the Hc-AFPs showed that most of the amino acid differences occurred in the α-helical regions of the peptides. By plotting the amino acid differences between the closely related Hc-AFP1 and 3 (94% similarity) where Ser17, Gly18 and Asn22 in Hc-AFP1 is replaced by Tyr17, Asp18 and Ser22 in Hc-AFP3 onto their respective models, it was observed that the change from a polar Gly18 to an acidic Aspartic18 residue in Hc-AFP3 resulted in a less polar α-helical region (Figure 4A and 4B). Root mean square deviation (RMSD) comparison between the structures of Hc-AFP1 and 3 revealed that these differences, although occurring in the α-helical region, caused a greater RMSD deviation in the N- and C-terminal ends of the peptide structure (Figure 4C). Comparative analysis of the amino acids sequences of Hc-AFP2 and 4 showed that they also share 94% similarity, with Asn19 and 27 (numbering according to Hc-AFP2) replaced with Gly19 and Arg27 and Lys30 replaced with Arg30 in Hc-AFP4 (Figure 4D and 4E). Comparative analysis of the structural models of Hc-AFP2 and 4 revealed that these amino acid changes had very little effect on the overall structure of these peptides and only had a RMSD difference of 0.26 Å in the α-helical region of the peptides (Figure 4F), leading to an extended α-helix in Hc-AFP2 when compared to Hc-AFP4 (Figure 4D and 4E). These amino acid substitutions did however result in a difference of the predicted surface properties between the Hc-AFP2 and 4 peptides. Hc-AFP4 is more basic and less hydrophilic in nature, whereas Hc-AFP2 is more polar in the regions surrounding the α-helix (Figure 4D and 4E).

The amino acids encoding for the α-helical region of Hc-AFP1 and 3 are unique when compared to defensins isolated from the other Brassicaceae species. Structural alignment of the backbones of the Hc-AFP1 - 4 models revealed that these unique amino acids present in the α-helical region of Hc-AFP1 and 3 (designated Group 1) resulted in a difference in tertiary structure when compared to Hc-AFP2 and 4 (designated Group 2) (Figure 4G). The α-helical regions of Group1 vs Group 2 had a RMSD value of more than 1.7 Å, and importantly, a significant difference of more than 1.6 Å was also observed in the Lβ_2_β_3 _loop, which is encoded by amino acids 38 to 41 (numbering according to Hc-AFP2) (Figure 4H).

### Expression analysis of the Hc-AFP encoding genes

**Figure 5:**
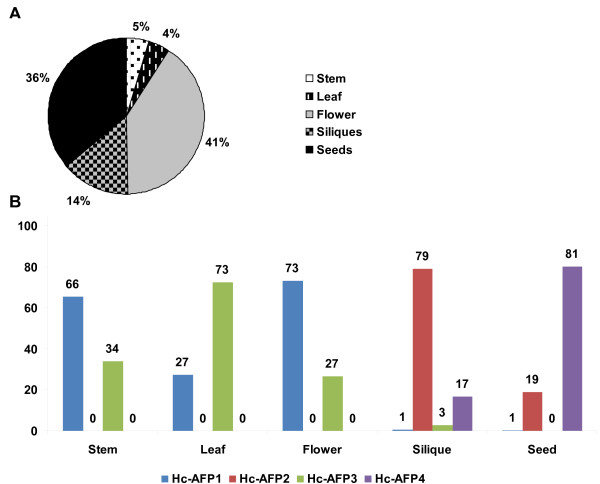
**The expression profile of the newly isolated defensin genes in the different tissue types of *H. coronopifolia *as determined with q-RT-PCR**. Data was analyzed using the software package LinRegPCR ver 11.0 and are expressed as an percentage of the total defensin transcript present at the moment of cDNA synthesis.

Quantitative RT-PCR (q-RT-PCR) analysis conducted on the *Heliophila *defensin encoding genes revealed that the reproductive and storage organs of the *H. coronopifolia *plant, which include the flowers, siliques and seeds, contributed to 91% of the observed defensin transcript present (Figure 5A). When considering the expression patterns of the individual peptides encoding genes, *Hc-AFP1 *and *3 *showed expression in vegetative and reproductive tissues tested (leaves, stems, flowers), as well as very low levels of expression in storage tissues (siliques and seeds) (Figure 5B). The distribution of these transcripts within the tissue types differed however, with *Hc-AFP1 *being the dominant transcript in stem and flower tissue contributing 66% and 73% respectively of the total defensin transcript present in these tissues. *Hc-AFP3 *was the dominant transcript in the leaf tissue contributing 73% of the observed defensin transcript present. In contrast *Hc-AFP2 *and *4 *were the dominant transcripts present in the storage organs of *H. coronopifolia *and not expressed in leaves, stems or flowers (Figure 5B). *Hc-AFP2 *was the dominant transcript in green siliques contributing 79% of the total defensin transcript present, but only contributed 19% of the total defensin transcript observed in mature seeds. *Hc-AFP4 *was predominantly expressed in seeds and to a much lesser extent in green siliques (Figure 5B).

### Bacterial production and purification of Hc-AFPs

**Figure 6:**
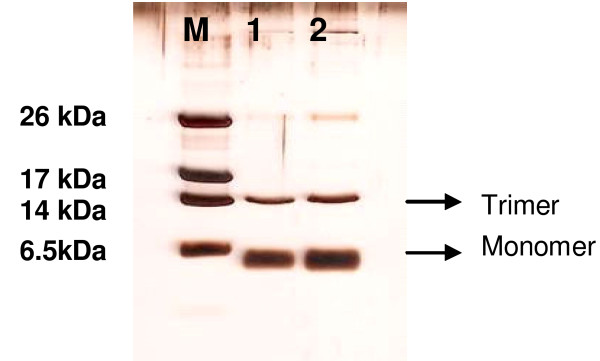
**Tris-Tricine SDS PAGE analysis of Hc-AFP2 and 4 (Lanes 1 and 2) eluted from the chitin bead column**. The Tris-Tricine SDS PAGE clearly shows the monomeric (5.7 kDA) and trimeric (15 kDa) form of Hc-AFP2 and 4. M = Low molecular weight marker.

The CBD-intein Hc-AFP fusions was successfully produced in *E. coli *strain BL21DE3 Rosetta gami pLysS and was visible as a 30 kDa band on a SDS PAGE gel (result not shown). The recombinant fusion proteins were successfully purified on a chitin bead column. On-column cleavage and peptide elution was confirmed with Tris-Tricine SDS PAGE analysis (Figure 6). The peptides were correctly folded, displaying the expected trimeric forms (15 kDa bands on the Tris-Tricine gel in Figure 6) of the defensin peptides.

Mass spectrometry analysis of the purified Hc-AFPs revealed molecular masses (in Dalton) of 5471.25 for Hc-AFP1, 5710.3 for Hc-AFP2, 5516.0 for Hc-AFP3 and 5724.4 for Hc-AFP4 respectively, which correlates with their predicted mono-isotopic masses calculated with the Expasy-Compute pI/Mw tool (Table 1) (-8 Da because of oxidized cysteines). This confirmed that the purified defensins were derived from their respective genes in the bacterial expression vectors and indicated that the crucially important four disulphide bridges common to all plant defensins peptides formed.

### Antifungal activity of the recombinant Hc-AFP peptides

The four plant defensin peptides from *H. coronopifolia *showed variable levels of activity against *B. cinerea *and *F. solani *in liquid plate assays (Table 2 and Additional File 1 and 2).

**Table 2:** Antifungal activity of the *Heliophila coronopifolia *defensins

	*Botrytis cinerea*				*Fusarium solani*			
**Defensin**	**IC_50_** **μg ml^-1^**	**Hyphal morphology**	**Spore lysis**	**MP^a^**	**IC_50_** **μg ml^-1^**	**Hyphal morphology**	**Spore lysis**	**MP^a^**

**Hc-AFP1**	> 25	Tip swelling	No	Yes	> 25	Mild hyper-branching	No	No

**Hc-AFP2**	10-15	Severe hyper-branching Tip swelling Lysis	Yes	Yes	10-15	Severe hyper-branching	No	Yes

**Hc-AFP3**	20-25	Severe hyper-branching Tip swelling and disruption	Yes	Yes	> 25	Mild hyper-branching	No	No

**Hc-AFP4**	15-20	Mild hyper-branching Tip swelling	No	Yes	5-10	Severe hyper-branching	No	Yes

^a^MP = Membrane permeabilization

Hc-AFP2 was the most active of all the peptides tested against *B. cinerea *with IC_50 _values ranging between 10-15 μg ml^-1 ^and a similar IC_50 _against *F. solani*. Hc-AFP4 inhibited *B. cinerea *with an IC_50 _value between 15-20 μg ml^-1^, and strongly inhibited *F. solani*, having an IC_50 _value ranging between 5-10 μg ml^-1 ^(Table 2 and Additional File 2).

**Figure 7:**
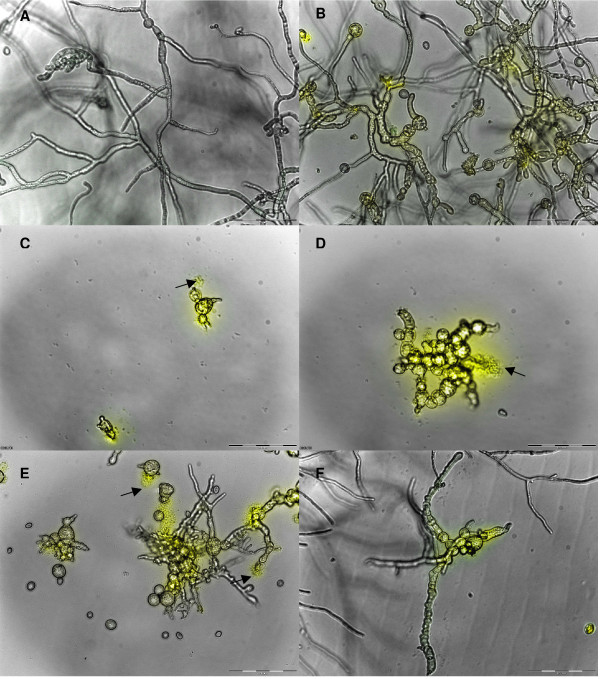
**Combined overlay of the light microscopical analysis at 20× magnification and the cell permeabilization assay conducted on *B. cinerea *grown in the presence of Hc-AFPs for 48 h at 23°C**. (**A**) Control, (**B**) Hc-AFP1 25 μg ml^-1^, (**C **and **D**) Hc-AFP2 15 μg ml^-1^, (**E**) Hc-AFP3 25 μg ml^-1^, (**F**) Hc-AFP4 18 μg ml^-1^. The yellow fluorescence indicates a compromised membrane and the black arrows indicate structures that are leaking their cellular content into the surrounding medium.

Microscopical analysis conducted on *B. cinerea *hyphae treated with the four defensin peptides revealed that all the defensins induced changes in *Botrytis *hyphal morphology under the conditions tested. Compared to the untreated control, hyper-branching, fungal tip swelling, increased granulation of hyphae and spores, as well as hyphal and spore disruption could be observed in the cultures treated with the peptides (Table 2 Figure 7 and Additional File 3). In addition, Hc-AFP2 and 3 had a severe effect on spore and hyphae integrity, resulting in disintegration of the hyphae and spores, which could be observed as leakage of the spore and hyphal cytoplasmic content into the surrounding environment. Moreover, assessment of propidium iodide assays revealed that the antifungal activity of all four *Heliophila *defensins against *B. cinerea *were associated with an increase in membrane permeabilization (Figure 7 and Additional File 3).

**Figure 8:**
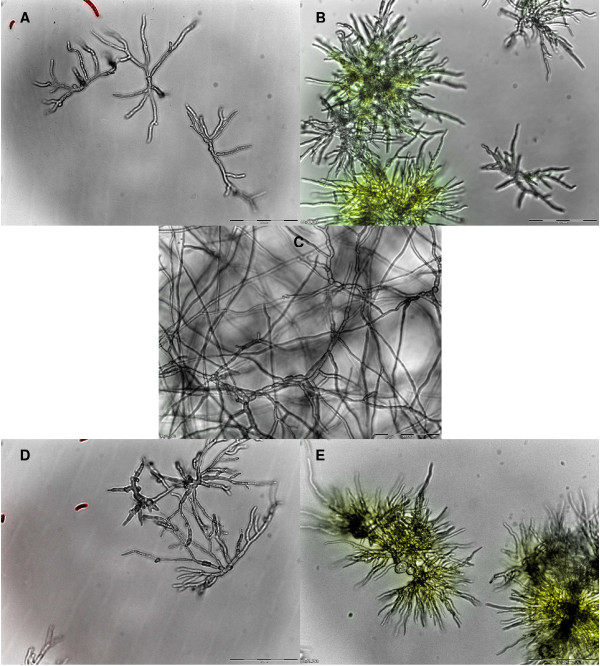
**Combined overlay of the light microscopical analysis at 20× magnification and the cell permeabilization assay conducted on *F. solani *grown in the presence of Hc-AFPs for 48 h at 23°C**. (**A**) Hc-AFP1 25 μg ml^-1^, (**B**) Hc-AFP2 12 μg ml^-1^, (**C**) Untreated control, (**D**) Hc-AFP3 25 μg ml^-1^, (**E**) Hc-AFP4 12 μg ml^-1^. The yellow fluorescence indicates a compromised membrane.

The peptides showed differential activity against *F. solani *(Table 2 Figure 8 and Additional File 4). Hc-AFP2 and 4 caused severe hyper-branching, as well as membrane permeabilization, whereas Hc-AFP1 and 3 caused mild hyper-branching and no membrane disruption against the wilting pathogen. Also, unlike the results on *Botrytis*, no lysis was observed in *F. solani *spores and hyphae when treated with the four plant defensins (Figure 8 and Additional File 4).

## Discussion

Plant defensins isolated from Brassicaceae species have especially shown great promise in the fields of agricultural biotechnology and therapeutic drug design. Several of these peptides have been overexpressed in crop species leading to disease resistant traits. The overexpression of BrD1, wasabi defensin and Rs-AFP2 have led to the engineering of disease resistant rice species [66,68,70,77], while the overexpression of AlfAFP1 yielded disease resistant potatoes at field trail level [65,82]. The overexpression of wasabi defensin in tomatoes also showed resistance towards necrotrophic pathogens [83]. Brassicaceae defensins are also used to evaluate the potential of defensin peptides in the design of new therapeutic drugs against human pathogenic yeast and fungi [62,63,84]. Moreover, since these defensins are well studied, they have been used as models to study the mechanisms of action of plant defensins against their target organisms [16,17,24,85-87]. Of the 449 defensin peptides listed in the protein database at the NCBI, 379 peptides belong to the Brassicaceae family.

Alignment analysis of the Brassicaceae defensin genes in the NCBI database revealed a high level of similarity (72%) in the first 20 bp that encode the start of the signal peptide (Additional File 5). By exploiting this homology, a PCR-based isolation strategy was used to amplify putative defensins from pools of cDNA made from the various tissue types of *H. coronopifolia*, a native South African Brassicaceae species currently unexplored for novel antimicrobial peptides. Four plant defensin peptide encoding genes, termed *Hc-AFP1 *to *4 *(Figure 1) were obtained and analysis of the deduced amino acid sequences revealed that the newly isolated peptide encoding genes shared the common structural design of other Brassicaceae defensins. Alignment analysis of the mature region showed that the Hc-AFP peptides shared 72% similarity at deduced amino acid level (Figure 3), and were more closely related to the defensins isolated from Brassicaceae species than from other plant species (Figure 2). Hc-AFP1 and 3 grouped closely together and displays amino acid sequences in the α-helix area unique to peptides in the Brassicaceae family. The homology models of the Hc-AFP peptides (Figure 4) revealed important differences between the different Hc-AFPs. Most of the amino acid differences occurred in the α-helical region, forming two structurally defined groups, with Hc-AFP1 and 3 in the first group and Hc-AFP2 and 4 in the second group. Despite the amino acid differences occurring in the α-helical region a large deviation were observed (1.7 Å) in the Lβ_2_β_3 _loop when the structures were superimposed (Figure 5G). Hc-AFP2 and 4 shares high homology to Rs-AFP2 and the Lβ_2_β_3 _loop of Rs-AFP2 have been well studied over the past years and have been linked to the antifungal activity of this peptide [16,86,87]. It was shown that the sequence ARHGSCNYVFPAHKCICYF is important for antifungal activity, especially the basic Arg32 residue and Tyr48 (numbering according to Rs-AFP2) [86]. This sequence is also present in the Hc-AFPs, but in Hc-AFP1 and 3 the important Arg32 is replaced by Ala, resulting in a less basic loop for Hc-AFP1 and 3 (charge at pH 7: +1.176) compared to Hc-AFP2 and 4 (charge at pH 7: +2.176). Recently it was shown that the overall charge of the Lβ_2_β_3 _loop (also termed the γ-core) is a determinant for the differential activities observed between closely related plant defensin peptides and might explain the differential antifungal activity observed between the Hc-AFP defensins [88]. The Lβ_2_β_3 _loop has also been connected with other biological activities associated with plant defensins, including anti insecticidal activity and enzyme inhibition [36,78]. The Lβ_2_β_3 _loop is not the only area of the peptide structure that plays a role in antifungal activity and recently a role for the loop connecting the α-helix and first β-strand have been proposed for the interaction of plant defensins with their fungal target [89,90], an area where the Hc-AFPs show high sequence divergence and a deviation of 1.6 Å when the structures are superimposed (Figure 5G and 5H).

### Expression profiling of the *Hc-AFP *genes

The differential and tissue-specific expression pattern of the *Heliophila *defensins proposes different roles for the four defensins. The expression of *Hc-AFP1 *and *3 *in the vegetative and floral tissues propose a role in the protection against fungal infection of these tissues. The significant contribution of *Hc-AFP1 *to the total pool of defensin transcripts present in the *H*. *coronopifolia *flowers might suggest a key role for *Hc-AFP1 *in the protection of the reproductive structure against pathogens. The very lytic activity of the peptides against *Botrytis *spores and hyphae might support this notion, since this necrotrophic pathogen typically attack vegetative and floral structures. Similarly, the strong activity against the wilting pathogen of the Hc-AFP2 and 4 peptides and their exclusive expression in the storage organs of the plant suggests that these peptides could be instrumental in protecting the germinating seeds against soil-borne pathogens such as *F. solani*. Moreover, the expression of the majority of *Heliophila *defensin transcript in the reproductive and storage organs is not unexpected, since the majority of isolated and characterized plant defensin peptides have been isolated from these organs [42,47-51,64,73,91-94], highlighting the importance of plant defensins in the protection of the reproductive systems of plants. This is especially well documented for the radish defensins Rs-AFP1 and 2, to which the *Heliophila *defensins share high homology. It has been shown that the radish defensins form preformed barriers within these tissues to stop the initiation or spread of fungal infection [42]. The tissue-specific expression of *Hc-AFP2 *and *4 *also propose a role in the protection of seeds against fungal attack as well as a possible role in protection during seed germination as has been observed for the radish defensins Rs-AFP1 and 2, which share 94% and 98% similarity to Hc-AFP2 and 4, respectively.

The differential expression pattern might, however, also indicate that the various peptides could play roles in the developmental and/or physiological processes of these organs and tissues, as was observed for some defensins isolated from maize and tomato [50,57]. These aspects need to be further evaluated with *in vivo *analysis.

### Recombinant production and purification

The high level of codon bias and the inability of *E. coli *to form disulphide bridges, solubility issues and affinity tag removal have made the production of plant defensins in bacteria notoriously difficult. By utilizing a codon-optimized *E. coli *strain with the ability to form disulphide bridges, we were able to successfully produce all four peptides in a soluble state.

The expression and purification strategy resulted in the purification (to homogeneity) of each peptide in a single chromatographic step. Disulphide bridge formation could also be confirmed by LC-MS analysis.

### Antifungal activity of the Hc-AFP peptides

Plant defensin peptides can be divided into three groups based on their antifungal activity. The first group known as morphogenic defensins are highly active against fungal pathogens and induce morphological changes in treated hyphae which results in severe hyper-branching of the fungal hyphae [14,21,71,95]. Most plant defensins isolated from Brassicaceae species belong to this group. The second group inhibits fungal pathogens, but do not induce morphological changes and are known as non-morphogenic defensins, with the third group not exhibiting any antifungal activity.

The peptides from *H. coronopifolia *were classified as morphogenic defensins since they had severe effects on hyphal development and morphology under the conditions tested. Recombinant Hc-AFP1 to 4 showed strong antifungal activity, also confirming the correct folding of the peptides during bacterial production. The peptides were tested against two agronomically important pathogens namely *B. cinerea*, the most destructive necrotrophic pathogen with a wide host range and the wilting disease agent *F. solani*. With the exception of Hc-AFP1, the Hc-AFPs showed strong activity against *B. cinerea *with IC_50 _values below 25 μg ml^-1^. All three peptides, with the exception of Hc-AFP1, induced a severe hyper-branching effect in treated hyphae, a common characteristic of defensins isolated from Brassicaceae species. The activity exerted by the Hc-AFPs against *B. cinerea *was also linked to membrane permeabilization, similar to what was observed for Rs-AFP2, a defensin from radish against fungal pathogens [96]. Hc-AFP2 and 3 had a severe effect on the integrity of *Botrytis *hyphae and spores, resulting in the disintegration of the fungal membrane and leakage of the cytoplasmic content into the surrounding environment. This lytic activity has not previously been described for Brassicaceae defensins according to our knowledge. The differential activity against *F. solani*, where Hc-AFP1 and 3 show reduced activity compared to Hc-AFP2 and 4, correlates well with their expression patterns. *F. solani *is a soil pathogen and Hc-AFP2 and 4, which show expression only in the storage organs, shows strong activity against this pathogen, strengthening the proposed role for these peptides during seed germination and seedling protection against soil borne pathogens. The expression of Hc-AFP1 and 3 in the vegetative tissues might also explain why they show more activity against pathogens evolved to infect vegetative tissues like the necrotrophic pathogen *B. cinerea*.

The effects on *Botrytis *(positive membrane disruption, severe morphological effects and even lytic activity) suggest that the activities could be orchestrated with the membrane being the primary target. However, recent evidence suggests that the cell wall also might play a role in membrane permeabilization [67] and that the membrane might actually be the secondary target. Interestingly, the *Fusarium *data indicated that Hc-AFP1 and 3 did not affect the membranes of the pathogen, since no membrane permeabilization was observed. These divergent activities of the *Heliophilia *peptides should be studied further. Future work will focus on exploring the structure-function relationships in the four peptides, and the implications on activity, specifically since these four peptides are highly homologous on amino acid sequence level, but display a few pointed changes in certain important defensin motifs which might be underpinning the observed variation in activities and mode-of action.

## Conclusions

The homology that resides within the signal peptides of plant defensins belonging to the same plant family is significant and allowed us to successfully implement a PCR-based method to isolate four Brassicaceae defensins. This strategy might be useful to isolate new defensin sequences from unsequenced plants species belonging to the same plant family. Despite the high level of homology on sequence level that was observed for the peptides, they were predicted to differ in their structural and surface properties, aspects that are known to influence activity levels and range. These aspects, as well as their observed divergent expression patterns, activities and modes of action against two test pathogens, provide interest to explore the structure-activity relationship of these peptides further.

## Methods

### Microbial strains and plant material

*Escherichia coli *strain DH5α were used for all cloning experiments, while *E. coli *strain BL21 Rosetta-gami pLysS DE3 (Novagen, Madison, WI, USA) were used for recombinant protein production. *Fusarium solani *and *Botrytis cinerea *cultures were obtained from the Department of Plant Pathology (DPP), Stellenbosch University and maintained on potato dextrose agar at 25°C until sporulation. Spores were harvested in dH_2_O and spore concentrations determined using a haemocytometer. *Heliophila coronopifolia *seeds were obtained from Silverhill seed company, South Africa. *H. coronopifolia *plants were established in potting soil from seeds and maintained under green-house conditions at 25°C.

### Design of Primer SPDEF-5'

The design of primer SPDEF-5 is based on the high level of homology that exists within the nucleotide sequences encoding for the signal peptides of plant defensin peptides belonging to the plant family Brassicaceae. Plant defensin encoding sequences isolated from Brassicaceae species was identified in the Genbank database of the National Centre for Biotechnological information (NCBI). The first 50 nucleotides encoding for the N-terminal signal peptide of the Brassicaceae plant defensin peptides were selected and aligned in AlignX (Invitrogen, Carlsbad, USA) (Additional File 5). 72% similarity existed over the first 50 amino acids. The consensus sequence were identified and the first 20 nucleotides were used to design primer SPDEF-5' (5'-ATGGCTAAGTTTGCTTCCATCAT-3').

### RNA isolation and cDNA synthesis

Total RNA was isolated from stem, leaf, flower, green siliques and mature seeds of *H. coronopifolia*. The tissue was ground to a fine powder in the presence of liquid nitrogen and total RNA was extracted from 200 mg powdered tissue according to Chang et al [97]. Total RNA was precipitated with 3 M LiCl and washed with 70% (v/v) ethanol and dissolved in 26 μl RNase free water. The samples were treated with DNaseI (Roche Diagnostics GmbH, Mannheim, Germany) to remove genomic DNA contamination in a 30 μl reaction. Sample volumes was adjusted to 200 μl with RNase free water and the DNaseI removed by extracting with an equal volume phenol/chloroform (50:50 v/v), followed by an equal volume chloroform to remove the phenol. The RNA was precipitated with a 1/10 volume 3 M NaOAc and 0.7 volumes isopropanol, washed with 70% (v/v) ethanol and dissolved in RNase free water. First strand cDNA was synthesized from 1 μg of total RNA using an anchored oligo dT_23 _primer (Sigma, St Louis, USA) and Superscript III (Invitrogen, Carlsbad, USA). cDNA synthesis was performed as described by the manufacturer.

### Gene isolation and cloning

The coding regions of potential plant defensin sequences were PCR amplified from total stem, leaf, flower, silique and seed cDNA using primer set SPDEF-5' and the anchored oligo dT_23 _primer (Sigma, St Louis, USA) used for cDNA synthesis. The PCR reaction was performed in a 50 μl reaction containing: 1× Expand buffer with 1.5 mM MgCl_2_, 0.2 mM dNTPs, 200 nM SPDEF-5' primer, 200 nM oligo dT_23_, 10 ng template DNA and 1 U Expand high fidelity polymerase (Roche Diagnostics GmbH, Mannheim, Germany). The PCR program was as follows: 95°C for 5 min; followed by 30 cycles of 95°C for 45 sec, 48°C for 30 sec and 72°C for 45 sec. PCR products were cloned into pGEM-T easy vector (Promega Corporation, Madison, USA) and positive clones were identified through restriction digest with *Eco*RI. Plasmids containing inserts were confirmed by sequencing. Obtained sequences were analyzed with the BLASTN algorithm http://blast.ncbi.nlm.nih.gov/Blast.cgi at the NCBI and clones containing open reading frames encoding for plant defensins were identified and termed pGEM-Hc1-4. The sequences were deposited to Genbank with the following accession numbers: JN203136 (Hc-AFP1), JN203137 (Hc-AFP2), JN203138 (Hc-AFP3) and JN203139 (Hc-AFP4).

### Bioinformatical analysis of the four *H. coronopifolia *defensin sequences

The deduced amino acid sequences of *Hc-AFP1*-*4 *was created in BioEdit [98] and analyzed with the Expasy-Compute pI/Mw tool http://web.expasy.org/compute_pi/ to obtain the different peptide parameters and Biochemistry online http://vitalonic.narod.ru/biochem to determine the overall charge of the peptides and their Lβ_2_β_3_-loops. The peptide structure of each peptide was evaluated for the presence of a signal peptide sequence with SignalP http://www.cbs.dtu.dk/services/SignalP/ and the possible disulphide bridge pattern for each peptide was determined using the web services DIpro http://download.igb.uci.edu/bridge.html.

The deduced amino acid sequences encoding for the mature plant defensin peptides were aligned against a diverse set of mature plant defensin sequences isolated from various plant genera. All sequences were obtained from the NCBI and alignment with the newly isolated defensins was performed in ClustalX [99]. A graphical representation of the phylogenetic tree was created in Arbodraw [100].

Homology models for each Hc-AFP peptide was created with the Bioinformatics toolkit at the Max Planck Institute for developmental biology http://toolkit.tuebingen.mpg.de/. The crystal structure of Rs-AFP1 (Protein Data Bank: 1AYJ) from radish was used as template. The models obtained were refined and analyzed with YASARA structure [101,102] and the FoldX plugin [103]. Models were visualized in Visual Molecular Dynamics ver 1.8.4 and the final images rendered with POV-Ray.

### q-RT-PCR analysis of the Hc-AFP encoding genes

Analysis was conducted on each of the newly isolated genes using the primer sets listed in Table 3. Each primer set was optimized to determine the optimal ratio of the forward and reverse primer in the primer set. The PCR efficiency was determined by setting up a standard curve prepared from the cDNA used to isolate the respective genes. The standard curve consisted of a 1/4 to 1/1024 dilution of the respective cDNA template. q-RT-PCR reactions with different ratios of forward and reverse primers were performed with the KAPA SYBR FAST qPCR Kit (Kapa Biosystems, South Africa) in a 20 μl reaction. All q-RT-PCR analysis were performed on a ABI7500 Real-Time PCR System (Applied Biosystems, South Africa) with the following program: 95°C for 5 min followed by 40 cycles of 95°C, 15 sec; 60°C, 32 sec. The 40 cycles was followed by a dissociation curve consisting of a ramp from 95°C to 60°C. The Ct values obtained were used as input data in the REST2009 software package [104] to calculate the PCR efficiency of each primer set. The optimized q-RT-PCR primer ratios were used to evaluate the expression of *Hc-AFP1*-*4 *in the different *H. coronopifolia *tissue types. The q-RT-PCR reactions were done as described above with each reaction containing the optimized primer concentration listed in Table 3. The data obtained were analyzed in LinRegPCR v11.0 software package [105] to determine the transcript levels for each gene present at the time of cDNA synthesis in the various tissue types. Elongation factor alpha (EFα) was used to standardize the expression levels obtained between the different tissues. The data for the individual genes are expressed as a percentage of the total defensin transcript present in the tissue.

**Table 3:** Primers used in the q-RT-PCR analysis of the Hc-AFP defensin genes

Primer set	Sequence 5'→3'	Primer	Target gene	**PCR eff**.
Helio EF Fw Helio EF Rv	ATGGGTAAAGAGAAGTTTCACATCAA GTTGGGTCCTTCTTGTCAACACTC	150 nm 200 nm	*H. coronopifolia *elongation factor 1α	0.99

Hc-AFP1 Rt Fw Hc-AFP1 Rt Rv	TCAGGAGTTTGTGGAAACAGTGG GCAGCCAACATAAACATATTTTGGA	200 nm 150 nm	*Hc-AFP1*	0.98

Hc-AFP2 Rt Fw Hc-AFP2 Rt Rv	CGTGTAGGAACCAGTGCATCAAC TAGGATTTTTCTGGTATGGCCG	150 nm 200 nm	*Hc-AFP2*	0.99

Hc-AFP3 Rt Fw Hc-AFP3 Rt Rv	TCAGGAGTTTGTGGAAACACTGA ATCATTAGAAGCTGCCAACATAAACTAG	150 nm 200 nm	*Hc-AFP3*	0.97

Hc-AFP4 Rt Fw Hc-AFP4 Rt Rv	ATGGTGGAAGCTCAGAAGTTGTGT GCTAGCAGCAAAGATGTTTGTTTG	200 nm 150 nm	*Hc-AFP4*	0.92

### Recombinant production of Hc-AFPs in *E. coli*

Hc-AFPs were produced in *E. coli *by using the IMPACT system (New England Biolabs, Ipswich, MA, USA). The DNA regions encoding for mature Hc-AFPs was cloned into the pTWIN1 vector, which allows for expression under control of the IPTG inducible T7 promoter. The cloning strategy allowed for a fusion between the Hc-AFPs and a chitin binding domain (CBD) to facilitate downstream purification using affinity chromatography. In the pTWIN system the Hc-AFPs and the CBD are separated by an intein peptide sequence that under goes self cleavage upon induction by pH and temperature shift.

The mature coding sequence of Hc-AFP1 to 4 was PCR amplified from pGEM-Hc1 to 4 using the primer sets listed in Additional File 6.

PCR reactions were performed in a 50 μl reaction volume containing: 1x Expand buffer with 1.5 mM MgCl_2_, 0.2 mM dNTPs, 200 nm Forward and Reverse primer, 1 ng template DNA and 1 U Expand high fidelity polymerase. The mature coding regions were PCR amplified using the following program, 95°C for 5 min; followed by 30 cycles of 95°C for 45 sec, 55°C for 30 sec and 72°C for 45 sec. PCR products were cloned into pGEM-T easy and positive clones were identified through digestion with *Eco*RI. Positive clones were termed pGEM-Hc1-Impact, pGEM-Hc2-Impact, pGEM-Hc3-Impact and pGEM-Hc4-Impact.

The mature coding regions were excised from their respective pGEM-Hc-Impact vectors with *Sap*I and *Pst*I and ligated into pTWIN1 vector prepared with *Sap*I and *Pst*I. Positive clones were identified by restriction digest and termed pTWIN-Hc1 to 4. All positive clones were sequenced with the SsPDnaB intein sequencing primer (5'-ACTGGGACTCCATCGTTTCT-3') to confirm the in-frame fusion between the CBD and the Hc-AFPs.

Recombinant production of the Hc-AFPs was performed in *E. coli *strain BL21DE3 Rosetta gami pLysS, which contains a plasmid encoding for 6 rare codons present in *E. coli*. pTWIN-Hc1 to 4 was transformed into the BL21 strain using a heat shock method and positive transformants were identified by plating onto LB agar plates containing 34 μg ml^-1 ^chloramphinicol, 12.5 μg ml^-1 ^tetracycline, 15 μg ml^-1 ^kanamycin and 100 μg ml^-1 ^ampicillin. Ten colonies of each construct were inoculated into a 5 ml preculture of LB broth containing the above mentioned antibiotics and incubated over night at 37°C. Four 2 L erlenmeyer flasks containing 400 ml LB broth plus antibiotics were inoculated with 1 ml preculture and incubated at 37°C with continuous shaking at 175 rpm. When the OD_600 _reached 0.7, the cultures were cooled to room temperature (22°C) and recombinant production of Hc-AFPs was induced with 0.4 mM IPTG (Roche Diagnostics GmbH, Mannheim, Germany). Recombinant production of Hc-AFPs was allowed to proceed for 6 hours at room temperature with continuous shaking at 175 rpm.

### Purification of Recombinant Hc-AFP defensins

Cells were collected from induced cultures by centrifugation. The cell pellet were resuspended in 40 ml cold column binding buffer (50 mM Tris-HCl pH 8.5, 1 M NaCl) supplemented with 5 mM MgCl and 0.2 mM PMSF (Roche Diagnostics GmbH, Mannheim, Germany). The cells were broken open by several cycles of freeze-thawing in liquid nitrogen and a 25°C water bath. The viscosity of the crude lysate was reduced by adding 50 units of DNaseI enzyme and incubation for 20 min at room temperature. The lysate was cleared of particulate matter by centrifugation at 10 000 rpm, at 4°C for 30 min.

Recombinant Hc-AFPs were purified using affinity chromatography. The cleared lysate was passed over a 100 x10 mm chitin bead column (New England Biolabs, Ipswich, MA, USA) equilibrated with column binding buffer at 4°C. The column was loaded using gravity flow and a reduced flow rate of 500 μl min^-1^. The column was washed with 200 ml of binding buffer at a flow rate of 3 ml min^-1^, followed by a quick flush of 20 ml cleavage buffer (200 mM NH_4_OAc pH 6.0). After the column was flushed with cleavage buffer, self cleavage of the intein peptide was induced by temperature shift to 30°C for 48 hours.

Cleaved peptide was eluted with 50 ml of cleavage buffer and freeze-dried. The freeze-dried peptide was subjected to a further two rounds of dissolving in 100 ml MilliQ water and freeze drying to remove most of the volatile ammonium salt. The peptide was finally dissolved in 2 ml MilliQ water followed by heat treatment at 80°C for 15 min to denature contaminant proteins. The sample was centrifuged at 12 000 rpm for 20 min and desalted on an Isolute C8 (EC) column (Biotage AB, Switzerland). The desalted peptide was eluted with 50% (v/v) acetonitrile and freeze-dried. Purified Hc-AFPs was dissolved in MilliQ water at a final concentration of 1 mg ml^-1^.

### Analysis and identification of recombinant Hc-AFP defensins

The purity of eluted Hc-AFPs was evaluated by separating 0.5 μg peptide on a 15% [w/v] Tris-Tricine gel [106]; after separation the peptide bands were visualized by silver staining.

Purified Hc-AFPs were subjected to LC-MS analysis to confirm that the plant defensins purified originated from their respective gene constructs. 10 μl purified Hc-AFP peptide was injected on a Waters Alliance 2690 Gradient UPLC and separated on a Waters UPLC BEH C18 column (2.1 × 50 mm, 1.7 μm) (Waters Corporation Milford MA, USA). The column was eluted with the program listed in Additional File 7. The eluted peak was submitted to MS analysis on a Waters API Q-TOF Ultima with the following settings: Source, ESI+; Capillary voltage, 3.5 kV; Cone voltage, 35; RF1, 40; Source, 100°C; Desolvation Temp, 350°C; Desolvation gas, 400 L h^-1 ^and Cone gas: 50 L h^-1^. The m/z ratios obtained were used to calculate the mono-isotopic mass of each peptide with all cysteine residues in an oxidized state. The mass obtained for each peptide was compared to the predicted mono-isotopic mass for each peptide generated with the Expasy-Compute pI/Mw tool (Table 1).

### Antifungal activity of Hc-AFPs

Quantitative antifungal activity of the Hc-AFPs was assessed using a microspectrophotometric assay [107]. The assay was performed in a 96-well microtiter plate (Bibby Sterilin Ltd, Stone, Staffs, UK), where each well contained 1000 fungal spores in 100 μl half strength Potato Dextrose Broth (PDB) and purified Hc-AFPs concentrations ranging from 5-25 μg ml^-1^. Control reactions contained no peptide. Plates were incubated in the dark at 23°C for 3 days, with microspectrophotometric readings taken every 24 hours at *A*_595_. Hc-AFP defensin activity was scored after 48 h and expressed in terms of % growth inhibition as described previously [107].

Microscopical analysis was conducted on *B. cinerea *grown in the presence of 25 μg ml^-1 ^Hc-AFP1 and 3, 15 μg ml^-1 ^Hc-AFP2 and 18 μg ml^-1 ^Hc-AFP4. *F. solani *was grown in the presence of 25 μg ml^-1 ^Hc-AFP1 and 3 and 12 μg ml^-1 ^Hc-AFP2 and 4. Microscopical assays were conducted in 200 μl reactions containing 1000 fungal spores in half strength PDB. After 48 h of growth at 23°C, the samples were treated with Anexin V and propidium iodide from an ApoAlert™ Annexin V Apoptosis Kit (Clonetech, Takara Bio Inc, Japan) before images were captured on an Olympus IX81 inverted microscope and analyzed with the CellIR^® ^software (Olympus Soft Imaging Solutions GmbH). Fluorescent images were captured with an intensity of 78% and an exposure time of 880 msec^-1^. Constant background subtraction, with a setting of 10, was performed on all captured images.

## Competing interests

The authors declare that they have no competing interests.

## Authors' contributions

MAV supervised the work and helped with conceptual design and manuscript preparation as well as final data analysis. AdB performed conceptual and experimental design and was responsible for all the research procedures, data analysis and writing the paper. Both authors read and approved the final manuscript.

## Supplementary Material

Additional File 1 **The antifungal activity of Hc-AFPs against *B. cinerea *after 48 h of growth at 23°C in the presence of 10-25 μg ml^-1 ^peptide**. Growth was monitored by measuring the absorption at 595 nm and compared to an untreated control. The data is presented as % growth inhibition as compared to the control reaction. (**A**) Hc-AFP1, (**B**) Hc-AFP2, (**C**) Hc-AFP3, (**D**) Hc-AFP4. Click here for file

Additional File 2 **The antifungal activity of Hc-AFPs against *F. solani *after 48 h of growth at 23°C in the presence of 10-25 μg ml^-1 ^peptide**. Growth was monitored by measuring the absorption at 595 nm and compared to an untreated control. The data is presented as % growth inhibition as compared to the control reaction. (**A**) Hc-AFP1, (**B**) Hc-AFP2, (**C**) Hc-AFP3, (**D**) Hc-AFP4. Click here for file

Additional File 3 **Combined overlay of the light microscopical analysis at 10× magnification and the cell permeabilization assay conducted on *B. cinerea *grown in the presence of Hc-AFPs for 48 h at 23°C**. (**A**) Hc-AFP1 25 μg ml^-1^, (**B**) Hc-AFP2 15 μg ml^-1^, (**C**) Untreated control, (**D**) Hc-AFP3 25 μg ml^-1^, (**E**) Hc-AFP4 18 μg ml^-1^. The yellow indicates a compromised membrane and clearly shows the leakage of the cellular content into the surrounding medium. Click here for file

Additional File 4 **Combined overlay of the light microscopical analysis at 10× magnification and the cell permeabilization assay conducted on *F. solani *grown in the presence of Hc-AFPs for 48 h at 23°C**. (**A**) Hc-AFP1 25 μg ml^-1^, (**B**) Hc-AFP2 12 μg ml^-1^, (**C**) Untreated control, (**D**) Hc-AFP3 25 μg ml^-1^, (**E**) Hc-AFP4 12 μg ml^-1^. The yellow indicates a compromised membrane. Click here for file

Additional File 5 **Alignment of the first 50 nucleotides of genes encoding for plant defensins belonging to the Brassicaceae family**. The high level of homology within the region encoding for the signal peptide was exploited to design primer SPDEF-5 (indicated in bold). Click here for file

Additional File 6 **Primers used in the construction of the bacterial expression vectors**. Click here for file

Additional File 7 **The elution program used on BEH C18 column during LC-MS analysis**. Click here for file
