# Supplementary material for: Four plant defensins from an indigenous South African Brassicaceae species display divergent activities against two test pathogens despite high sequence similarity in the encoding genes
Source: BMC Res Notes. 2011 Oct 28;4:459. doi: 10.1186/1756-0500-4-459 (PMC3213222; doi:10.1186/1756-0500-4-459)
Supplement: Additional File 2 — The antifungal activity of Hc-AFPs against F. solani after 48 h of growth at 23°C in the presence of 10-25 μg ml-1 peptide. Growth was monitored by measuring the absorption at 595 nm and compared to an untreated control. The data is presented as % growth inhibition as compared to the control reaction. (A) Hc-AFP1, (B) Hc-AFP2, (C) Hc-AFP3, (D) Hc-AFP4. [file 1756-0500-4-459-S2.DOC]

**A**

**B**

**C**

**D**
