# Supplementary figures and images for: Four plant defensins from an indigenous South African Brassicaceae species display divergent activities against two test pathogens despite high sequence similarity in the encoding genes
Source: BMC Res Notes. 2011 Oct 28;4:459. doi: 10.1186/1756-0500-4-459 (PMC3213222; doi:10.1186/1756-0500-4-459)

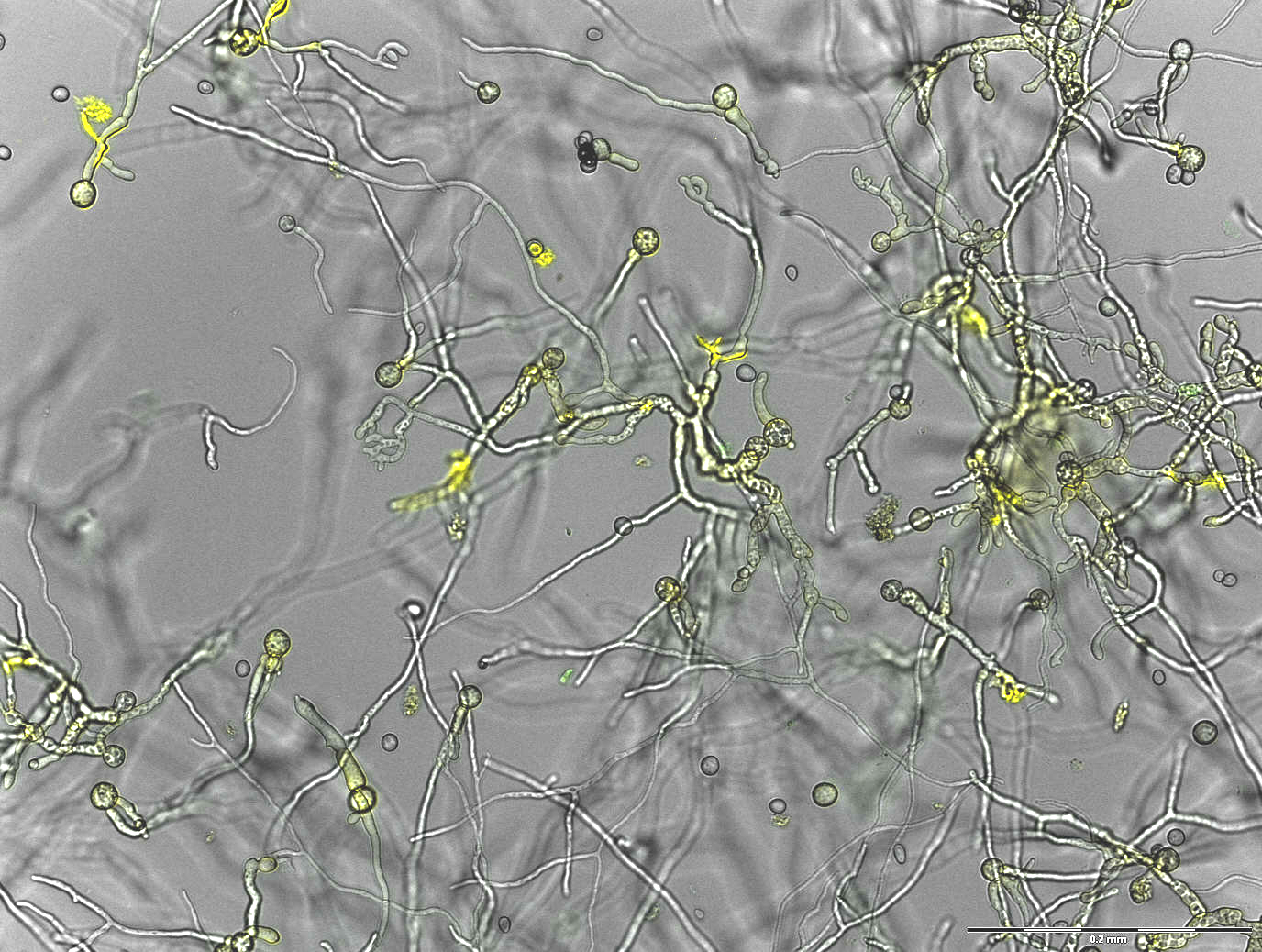

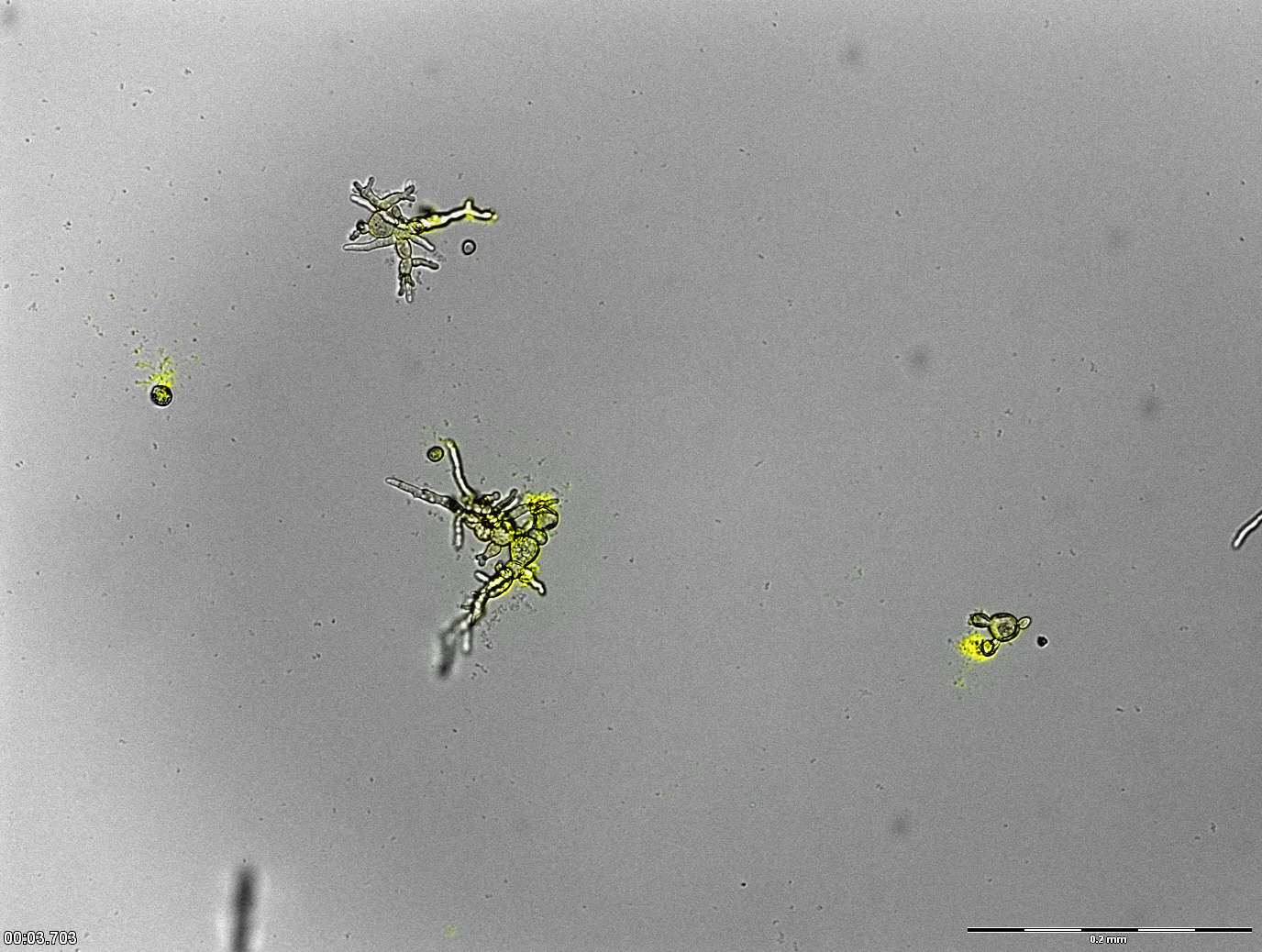


**A**

**B**


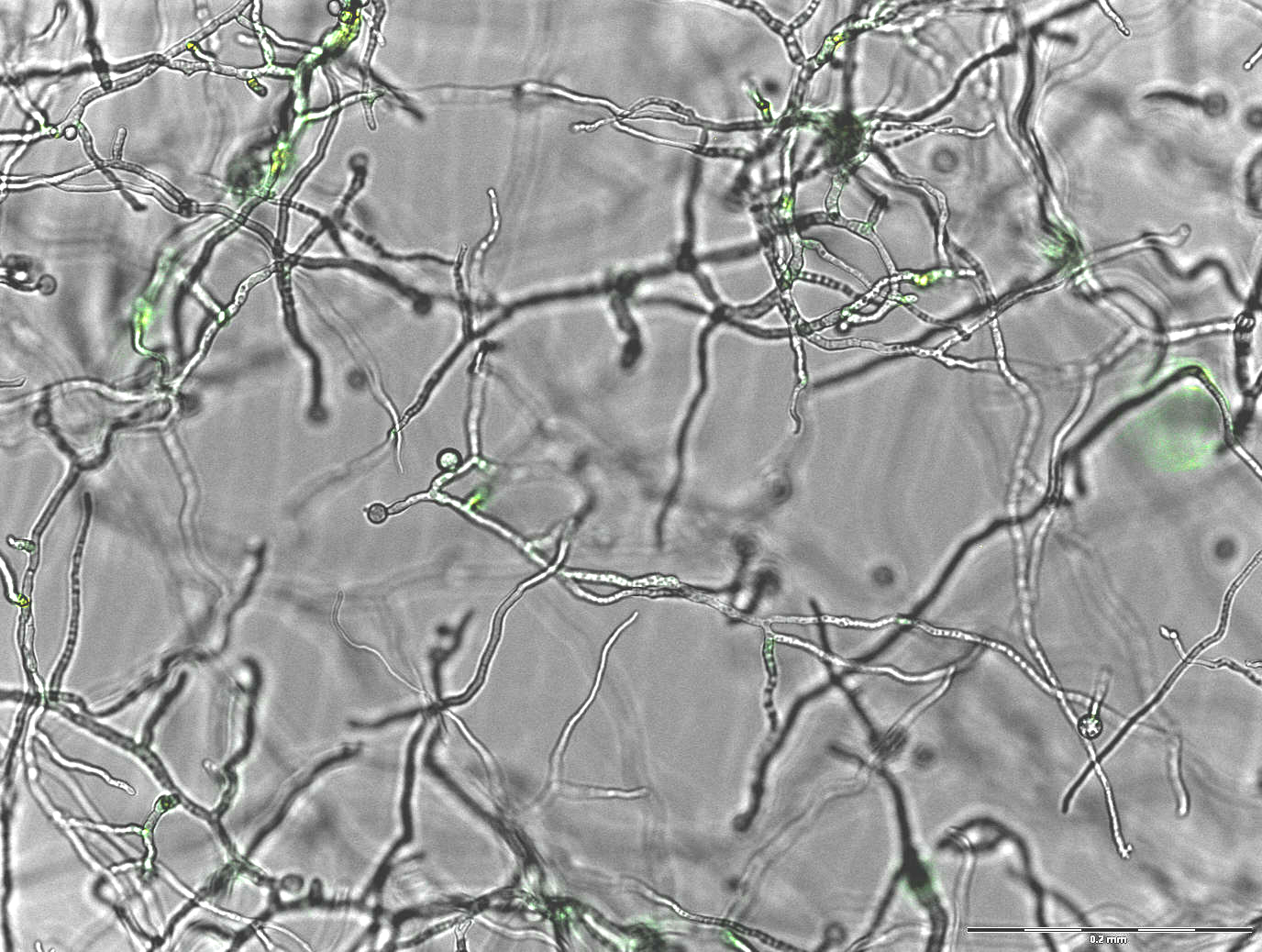


**C**


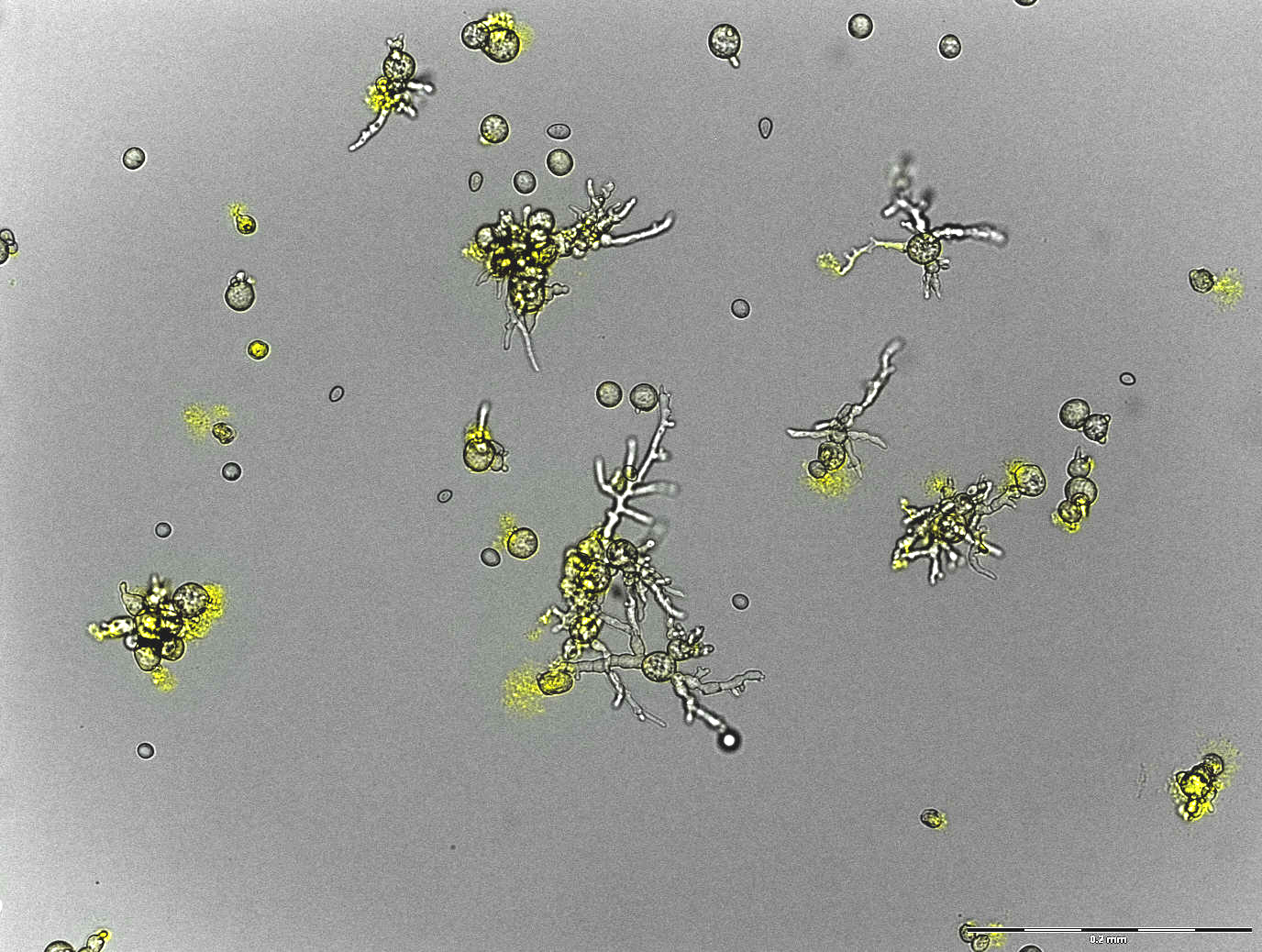

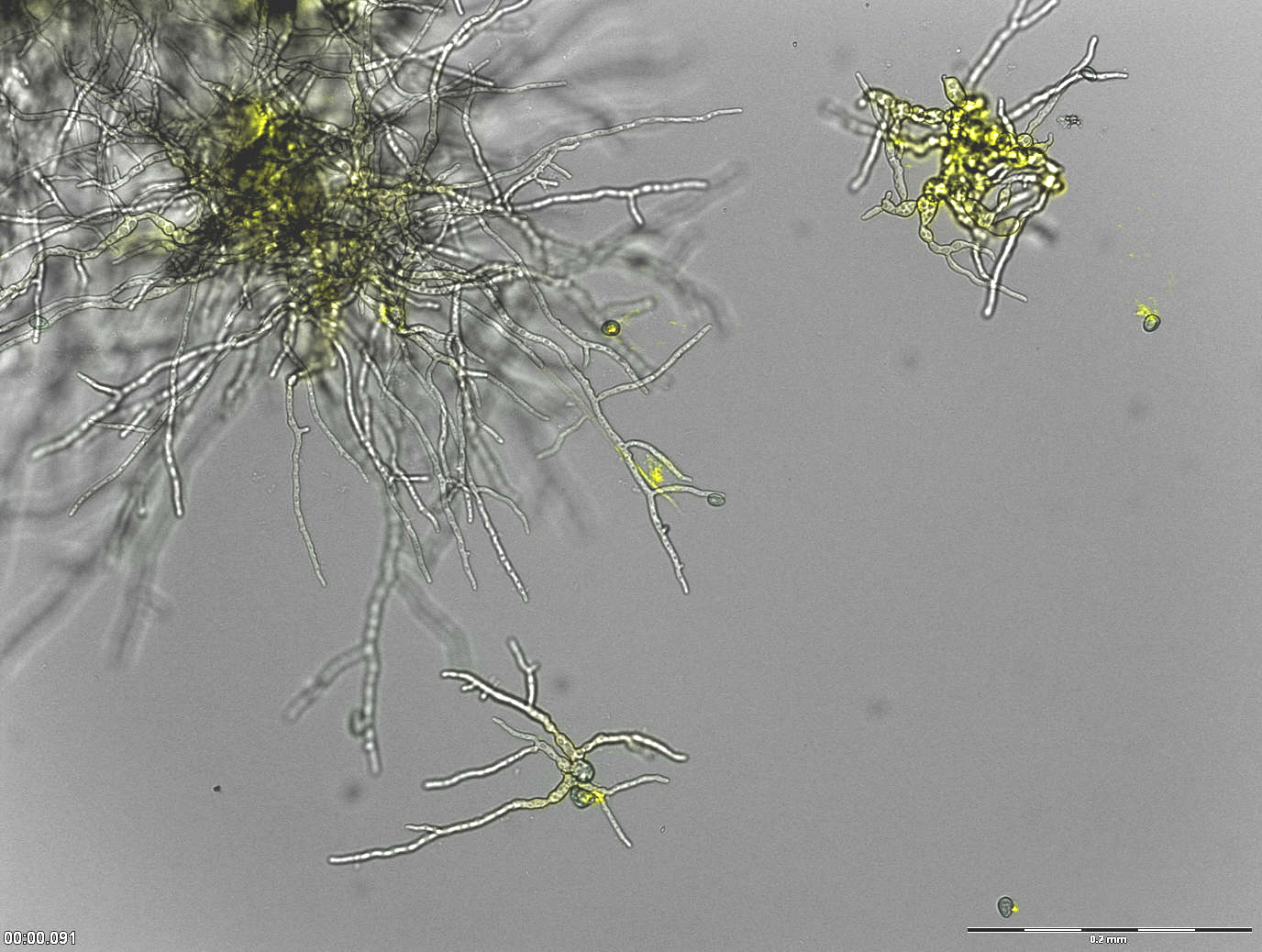


**D**

**E**

Supplement: Additional File 3 — Combined overlay of the light microscopical analysis at 10× magnification and the cell permeabilization assay conducted on B. cinerea grown in the presence of Hc-AFPs for 48 h at 23°C. (A) Hc-AFP1 25 μg ml-1, (B) Hc-AFP2 15 μg ml-1, (C) Untreated control, (D) Hc-AFP3 25 μg ml-1, (E) Hc-AFP4 18 μg ml-1. The yellow indicates a compromised membrane and clearly shows the leakage of the cellular content into the surrounding medium. [file 1756-0500-4-459-S3.DOC]

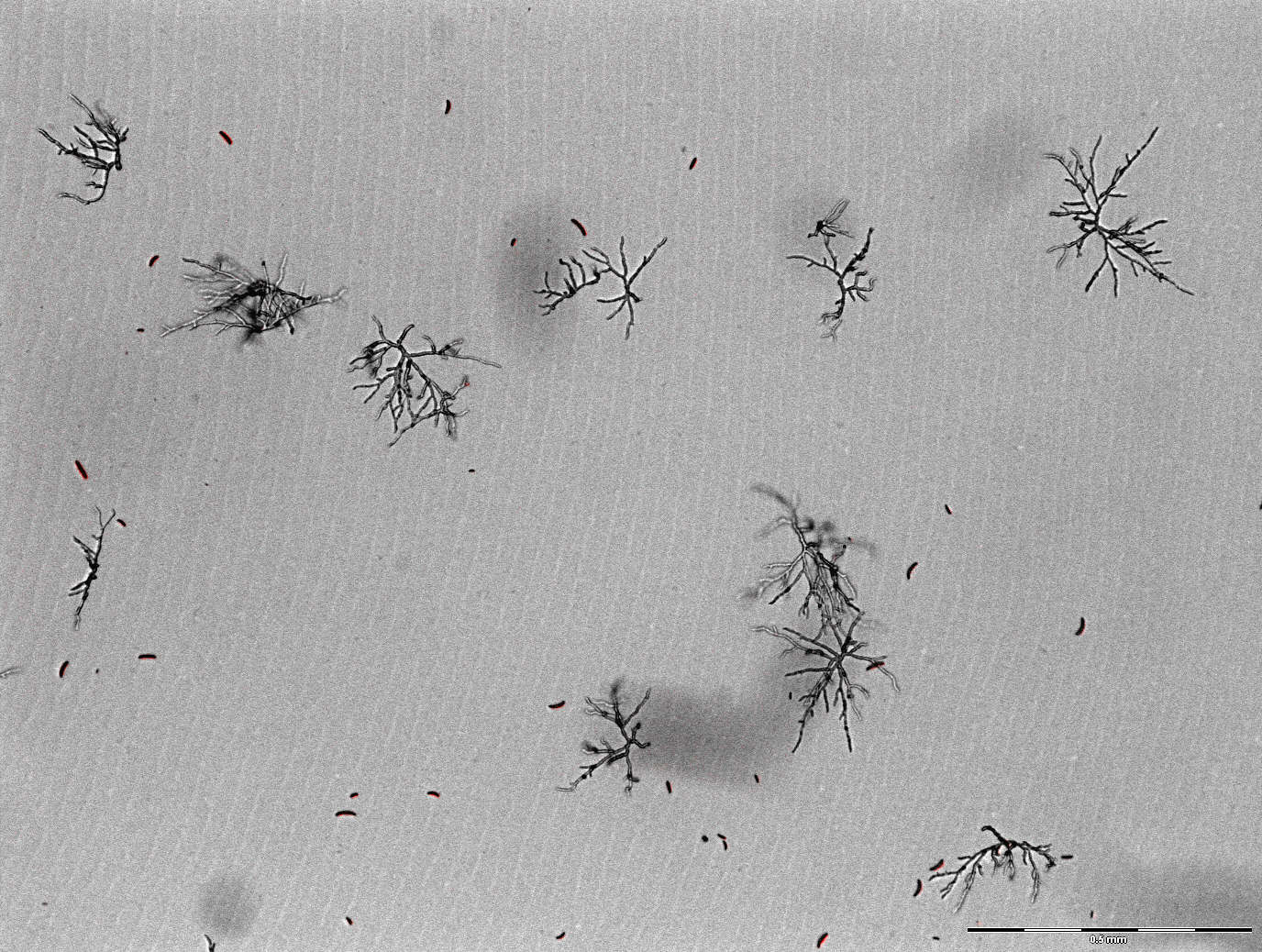

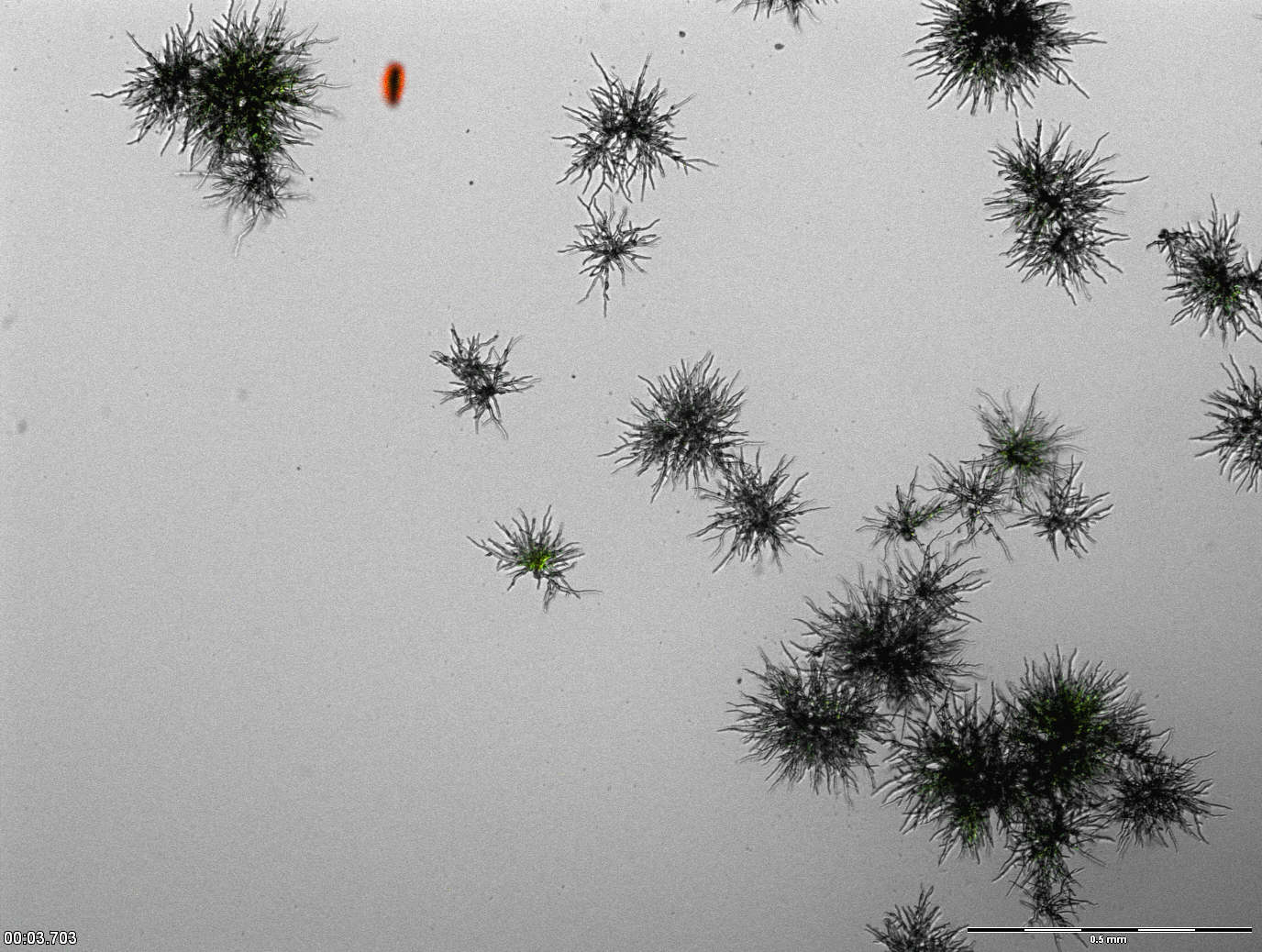


**A**

**B**


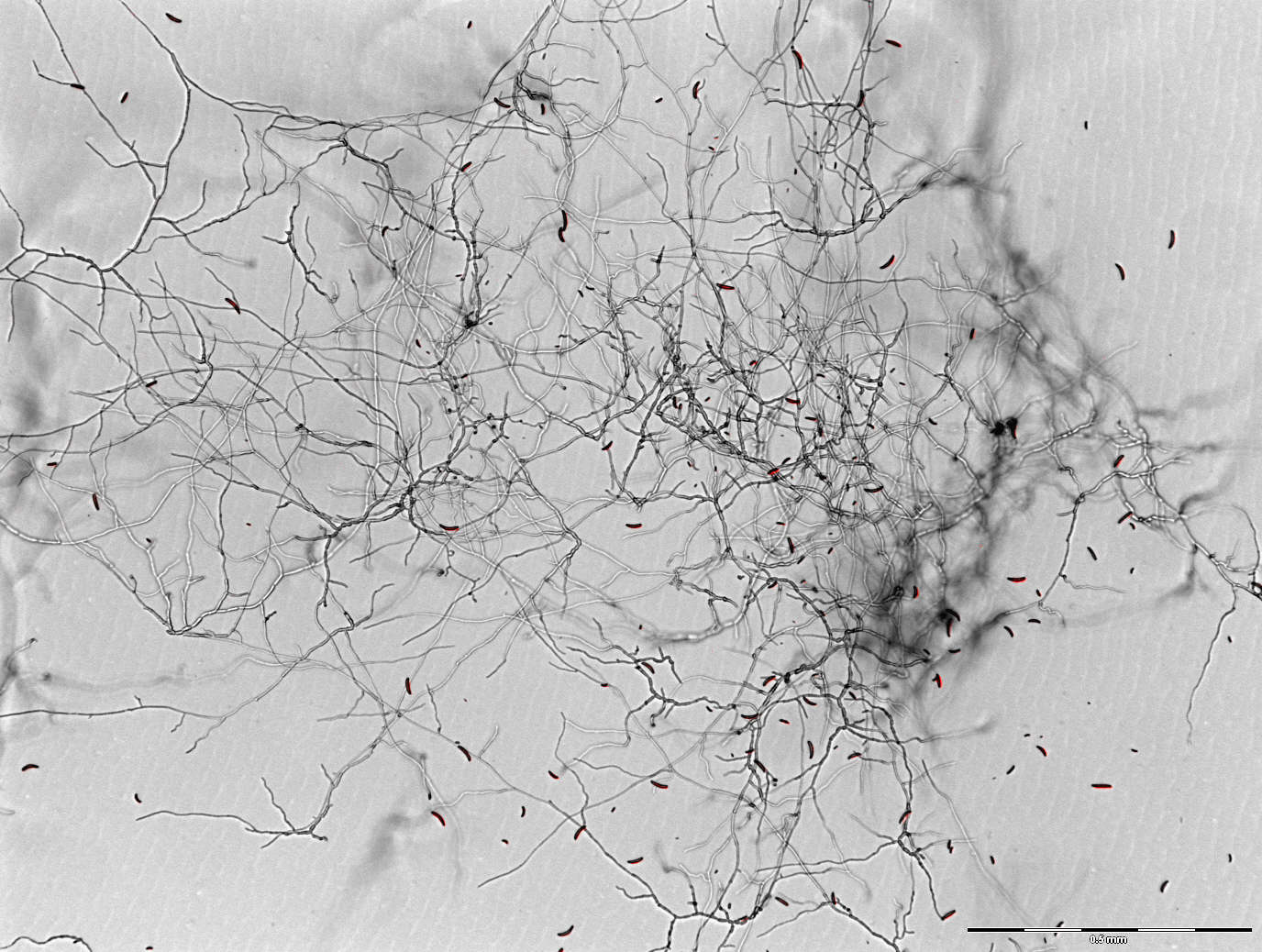


**C**


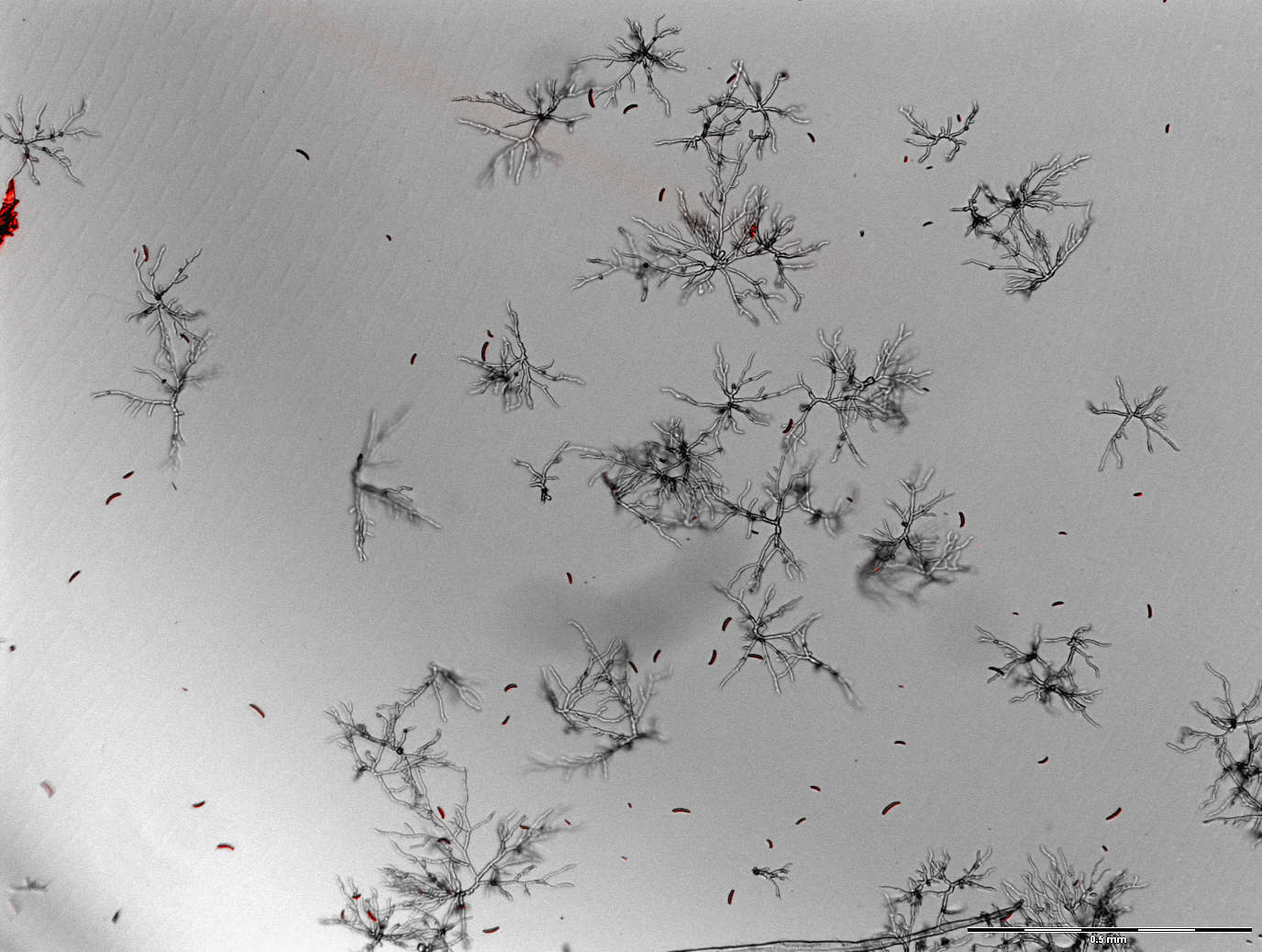

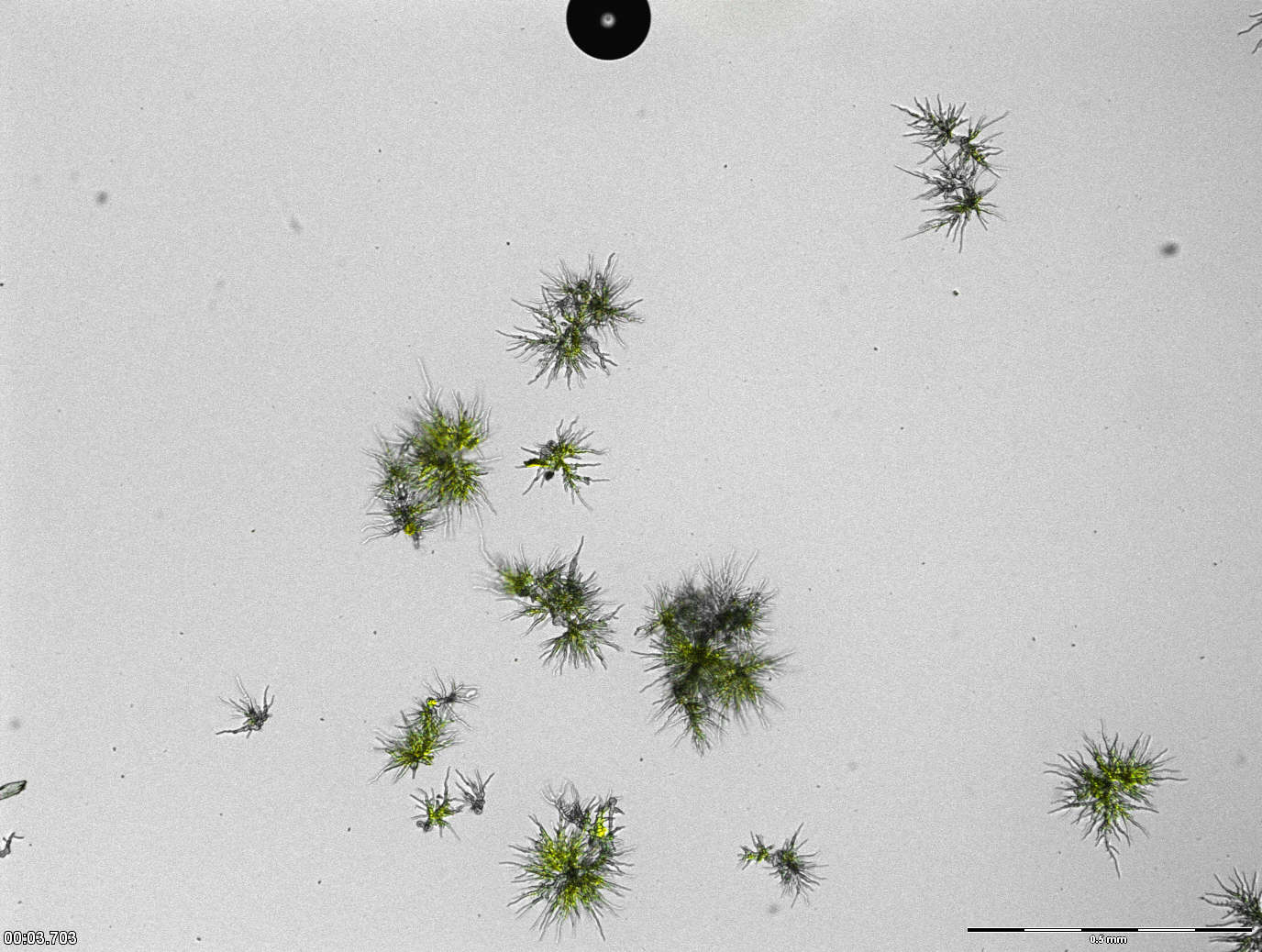


**D**

**E**

Supplement: Additional File 4 — Combined overlay of the light microscopical analysis at 10× magnification and the cell permeabilization assay conducted on F. solani grown in the presence of Hc-AFPs for 48 h at 23°C. (A) Hc-AFP1 25 μg ml-1, (B) Hc-AFP2 12 μg ml-1, (C) Untreated control, (D) Hc-AFP3 25 μg ml-1, (E) Hc-AFP4 12 μg ml-1. The yellow indicates a compromised membrane. [file 1756-0500-4-459-S4.DOC]
