# Supplementary material for: Four plant defensins from an indigenous South African Brassicaceae species display divergent activities against two test pathogens despite high sequence similarity in the encoding genes
Source: BMC Res Notes. 2011 Oct 28;4:459. doi: 10.1186/1756-0500-4-459 (PMC3213222; doi:10.1186/1756-0500-4-459)
Supplement: Additional File 5 — Alignment of the first 50 nucleotides of genes encoding for plant defensins belonging to the Brassicaceae family. The high level of homology within the region encoding for the signal peptide was exploited to design primer SPDEF-5 (indicated in bold). [file 1756-0500-4-459-S5.DOC]

1 50

gi_11691893_dbj_AB012871.1_ (1) ATGGCTAAGTTTGCTTCTATCATCGCTCTTCTCTTCGCTGCTCTTGTTCT

gi_242724020_gb_FJ489240.1_ (1) ATGGCTAAGTTTGCTTCCATCACCGCCCTTCTCTTTGCTGCTCTTGTTCT

gi_13872713_emb_AJ311046.1_ (1) ATGGCTAAGGTTGCTTCCATCGTTGCCCTTCTTTTTCCTGCCCTTGTTAT

gi_81176558_gb_DQ191751.1_ (1) ATGGCTAAGGTTGCTTCCATCATTGCCCTACTTTTTGCTGCTCTTGTTCT

gi_81176560_gb_DQ191752.1_ (1) ATGGCTAAGGTTGCTTCCATCATTGCCCTACTTTTTGCTGCTCTTGTTCT

gi_63029686_gb_AY961379.1_ (1) ATGGCTAAGTCTGCTACCATCGTTACCCTTTTTTTCGCTGCTCTTGTTTT

gi_56385121_gb_AY829229.1_ (1) ATGGCTAAGTTTGCTTCTATCGTTCCCCTTCTCTTCGCTGCTCTTCTTCT

gi_23321204_gb_AF528180.1_ (1) ATGGCTAAGTTTGTTTCCATCATCACCCTTTTCTTCGCTGCTCTCGTTCT

gi_37528804_gb_AY383485.1_ (1) ATGGCTAAGTTTGTTTCCATCATCACCCTTCTCTTCGCTGCTCTCGTTCT

gi_281486467_gb_GU230169.1_ (1) ATGGCTAAGGTTGCTTCCATCATCACCCTTCTCTTCGCTGCTCTCGTTCT

gi_63029680_gb_AY961376.1_ (1) ATGGCTAAGTTTGCTTCCATCATCACCCTTATCTTCGCTGCTCTTGTTCT

gi_63029682_gb_AY961377.1_ (1) ATGGCTAAGTTTGCTTCCATCATCACCCTTATCTTCGCTGCTCTTGTTCT

gi_63029684_gb_AY961378.1_ (1) ATGGCTAAGTTTGCCTCCATCATCACCCTTATCTTCGCAGCTCTTGTTCT

gi_151382078_gb_EF423802.1_ (1) ATGGCTAAGTTTGCTTCCATCATCACCCTTCTCTTCGCTGCTCTTGTTGT

gi_218436743_dbj_AB302324.1_ (1) ATGGCTAAGTTTGCTTCCATCATCACCCTTCTCTTCGCTGCTCTTGTTGT

gi_244539518_dbj_AB302891.1_ (1) ATGGCTAAGTTTGCTTCCATCATCACCCTTCTCTTCGCTGCTCTTGTTGT

gi_60686418_gb_AY884023.1_ (1) ATGGCTAAGTTTGCTTCCATCATCACCCTTCTCTTCGCTGCTCTTGTTGT

Consensus (1) **ATGGCTAAGTTTGCTTCCAT**CATCACCCTTCTCTTCGCTGCTCTTGTTCT
