# Supplementary material for: Four plant defensins from an indigenous South African Brassicaceae species display divergent activities against two test pathogens despite high sequence similarity in the encoding genes
Source: BMC Res Notes. 2011 Oct 28;4:459. doi: 10.1186/1756-0500-4-459 (PMC3213222; doi:10.1186/1756-0500-4-459)
Supplement: Additional File 6 — Primers used in the construction of the bacterial expression vectors. [file 1756-0500-4-459-S6.DOC]

| **Primer set** | **Sequence** | **Target Gene** | **Template** |
| --- | --- | --- | --- |
| Hc‑AFP1/3-ImpactF  Hc-AFP1/3 ImpactR | GGTTGCTCTTCCAACAGGTACTGTGAGAGATCGAGT  CGGCTGCAGTTAACATGGGTAGTAACAGA | *Hc-AFP1*  *Hc-AFP3* | pGEM-Hc1  pGEM-Hc3 |
| Hc-AFP2 ImpactF  Hc-AFP2 ImpactR | GGTTGCTCTTCCAACCAAAAGTTGTGTGAGAGACCA  CGGCTGCAGTTAACATGGGAAGTAGCAGA | *Hc-AFP2* | pGEM-Hc2 |
| Hc-AFP4 ImpactF  Hc-AFP4 ImpactR | GGTTGCTCTTCCAACCAGAAGTTGTGTGAGAGACC  CGGCTGCAGTTAACATGGGAAGTAACAGA | *Hc-AFP4* | pGEM-Hc4 |
