# Supplementary material for: Four plant defensins from an indigenous South African Brassicaceae species display divergent activities against two test pathogens despite high sequence similarity in the encoding genes
Source: BMC Res Notes. 2011 Oct 28;4:459. doi: 10.1186/1756-0500-4-459 (PMC3213222; doi:10.1186/1756-0500-4-459)
Supplement: Additional File 7 — The elution program used on BEH C18 column during LC-MS analysis. [file 1756-0500-4-459-S7.DOC]

| **Time (min)** | **Flow Rate** | **%A (0.1% formic acid)** | **%B (acetonitrile)** | **Curve** |
| --- | --- | --- | --- | --- |
| 1. Initial | 0.400 | 90.0 | 10.0 |  |
| 2. 0.10 | 0.400 | 90.0 | 10.0 | 6 |
| 3. 3.00 | 0.400 | 0.0 | 100.0 | 6 |
| 4. 5.00 | 0.400 | 0.0 | 100.0 | 6 |
| 5. 6.00 | 0.400 | 90.0 | 10.0 | 6 |
| 6. 7.00 | 0.300 | 90.0 | 10.0 | 6 |
